# Innovation, Demand, and Responsibility: Some Fundamental Questions About Health Systems: Comment on "What Health System Challenges Should Responsible Innovation in Health Address? Insights From an International Scoping Review"

**DOI:** 10.15171/ijhpm.2019.47

**Published:** 2019-07-06

**Authors:** Harro van Lente

**Affiliations:** Faculty of Arts and Social Sciences, Maastricht University, Maastricht, The Netherlands.

**Keywords:** Health System, Innovation, Demand, Responsible Innovation

## Abstract

In this commentary on the exercise of Lehoux et al (this volume) I argue that in discussions on the current challenges of health systems, a better diagnosis of the health system is required. The cause of responsible innovation in health (RIH) requires a better understanding of the dynamics of health systems, in particular how innovation, demand, and responsibility are manifested. Innovation brings its own dynamic to the health system; demands are linked to historical and social developments; responsibility brings contestations about what counts as good healthcare. Any attempt of RIH should include such reflections.

Everywhere in healthcare, questions of future directions are prominent. A key question is what today’s urgent healthcare problems are and how they should be addressed by health systems – a question that is addressed by Pascal Lehoux and colleagues (this volume).^1^ In their paper, they map the current challenges of health systems through an extensive global literature review; they also argue that addressing these challenges should be on the agenda of responsible innovation in health (RIH). Their exercise is rigorous, timely and a valuable landmark for health policy.

The appeal to guide the health system towards more responsibility and sustainability is appropriate, yet seems to ignore some pertinent questions about what health systems are and do. The assumption seems to be that once priorities are known, policy-makers are able to guide the health systems into the desired direction. Of course, no one will deny that other factors play a role as well, sometimes referred to as ‘politics’ or ‘opposition.’ It is helpful, however, to pay some more careful attention to these other factors. In fact, the exercise of Lehoux and colleagues itself points to some fundamental issues about the dynamics of health systems. A good starting point is their observation at the beginning of their Concluding Remarks section: “Since the late 1980s, new health technologies not only increased global inequalities, but they also undermined the sustainability of health systems in rich and poor countries alike.” In other words, more investments, intensified efforts and better technologies do not just bring progress, they may also bring detrimental consequences. This dramatic observation is countered by Lehoux et al by the urge to do better: “It will be imperative to implement policy mechanisms that can support the development, financing and use of innovations that do not compromise but rather contribute to the success and sustainability of health systems.” I would argue that this urge to do better is not sufficient: we also need a better *diagnosis* of why health systems may not deliver their promises. In this commentary, I will address some fundamental issues about the dynamics of health systems, in particular in relation to the key notions in the paper of Lehoux et al: innovation, demand, and responsibility.

## Innovation

In health systems, technologies obviously play a central role and using and improving them (innovation) often makes sense. Due to new technologies, health systems are better able to monitor patients, deliver drugs and facilitate care. Yet, as a rule, technologies are not simply clever answers to good questions; they bring about novel dynamics, too. I will support my argument with the example of the introduction of the Da Vinci robot in surgery in the Netherlands, studied by Abrishami and colleagues.^2^ Their study shows that decisions to install a very expensive Da Vinci surgical robot have not been the result of clear cost-benefit calculations, or by health technology assessment reports, but are the outcomes of complex interactions and interdependencies. Hospitals, for instance, see the purchase of a Da Vinci surgical robot as a move in their ongoing competition for status, reputation and visibility with other hospitals. Patients, in their turn, want the best treatment possible and take ‘novel’ as an indication with ‘better,’ hence express a favour for robotized surgery. Policy-makers, on the other hand, follow the choice of patients, believing that ‘the market’ will reduce costs and increased efficiency. Surgeons seek to reaffirm their pivotal role in health systems, while elder surgeons who suffer a bit more from trembling than in their younger days favour the surgical robot to hide their declining skills. The innovation of the surgical robot, thus, brings much more to the table than just technical gains – something to be taken on board by policy-makers.

## Demand

A second fundamental issue concerns the idea of demand. The mapping of demands that Lehoux et al perform is based on the assumption that this is a useful starting point for innovation – a contested assumption. Some decades ago, the discussion about the drivers of innovation was phrased in terms of ‘demand pull’ versus ‘technology push’: does the origin of novelty reside in what people need, with innovation as a response, or does it start with new technologies inciting use and further adoption? The consensus in innovation studies now is that both mechanisms are at work, but that the dichotomy is misleading, too, as both extremes presume a linearity in the development from invention on the one hand, to usage at the other. Many studies have shown that inventions are shaped by interests and imaginations, while usage includes more than a passive uptake.^3^ Users are innovators in their own right.

What is more, demand in health systems is never just a simple expression of what is needed, but also an expression of what can be rightfully asked for. Demand, thus, also expresses competition, imitation and cultural shifts in what counts as ‘healthy’ and ‘justified.’ For example, in many countries in vitro fertilization is now seen as a proper response to a rightful demand; this was certainly not the case several decades ago, when the mere possibility of in vitro fertilization entered the world.^4^ At that time it was seen as unnatural and beyond the tasks of health systems.

My point is not to be cynical or relativistic about demands in health systems – the suffering is real and the need to respond is urgent – but to fully acknowledge that demand is not ‘just there,’ waiting to be mapped. Demand is always an outcome of societal developments with a history, a context with winners and losers; it is a summary of ideas about what needs to be repaired, about possibilities to do so and about the justification to perform. In this way, also the issue of rising costs of healthcare due to rising demand should also be interpreted differently. Instead of understanding the rise of healthcare costs as a burden to be alleviated with austerity, one could also interpret the rising costs as an indication of changing possibilities and priorities: apparently, a society is able and willing to spend more on healthcare.

## Responsibility

Lehoux et al see RIH as an endeavour to guide health systems towards what is ‘affordable,’ ‘acceptable,’ and ‘adaptable.’ Their characterization of responsibility resonates with the rise of responsible innovation in research agendas at large, such as those in European Horizon 2020 programmes. It also reflects a broader tendency in science, technology and health policies to guide innovations towards societal needs. Clearly, the notion of responsibility comes with high hopes that it will give directions for further (health) research. Yet, these directions will not come from formulating the right principles alone – it will also necessarily involve negotiation, articulation, and contestation.

A strong example of such struggles around ‘responsible’ healthcare is the case of early diagnostics for Alzheimer’s disease.^5^ Clearly, Alzheimer’s disease is one of the major global challenges, in particular in higher income countries. As a response, medical researchers make huge efforts to study the disease and receive large funds to diagnose it earlier, in the hope to unravel the pathways of the disease. These research directions can be questioned in many ways. For instance, what is being diagnosed in the first place? The more research publications on the condition appear, the more the notion of Alzheimer’s disease itself becomes elusive. The clinical indications vary enormously and follow many tracks such as the significance of amyloid proteins in the brain, or the consequences of ‘tangles.’ The scientific disputes even extend to the question whether it should be counted as a disease at all, or, as some geriatrists argue, as a ‘natural’ aging of the body and the brain. Another critique on the massive investments into research and the concomitant hopes of cure is that these go at the expense of other forms of care.^6^ Many argue that there are better ways to take care of the millions of patients and caretakers that currently suffer from the disease. Some even argue that it is not ethical to launch promises about an eventual cure. Typically, medical research funding comes with a discourse of hope and fear, so-called regimes of hope, which may indeed lead to funding but will also feed concerns and existential fears.^7^

What the example makes clear is that when considering responsible innovations in health systems, more is at stake than a decision to fund or not to fund. Is it justified, for instance, to focus on early diagnosis, in particular when a cure is not in sight, or is it more responsible to invest in better forms of care? What are the consequences of launching promises of early diagnosis and of a possible cure in the distant future? Such questions require negotiation, articulation and any answer can be contested, in principle and in practice.

The three fundamental questions about innovation, demand, and responsibility show that a health system is more than a collection of care practices supported with technologies. It entails also collective hopes of what can be achieved, historically informed imaginations of what is healthy and justified and contested ideas of what is responsible. Any attempt for a responsible health system, thus, implies a further diagnosis of how health systems work and a collective reflection on what directions are desirable and responsible.

## Ethical issues

Not applicable.

## Competing interests

Author declares that he has no competing interests.

## Author’s contribution

HvL is the single author of the paper.

## References

[1] Lehoux P, Roncarolo F, Silva HP, Boivin A, Denis JL, Hébert R (2019). What health system challenges should responsible innovation in health address? Insights from an international scoping review. Int J Health Policy Manag.

[2] Abrishami P, Boer A, Horstman K (2014). Understanding the adoption dynamics of medical innovations: affordances of the da Vinci robot in the Netherlands. Soc Sci Med.

[3] Hyysalo S, Jensen TE, Oudshoorn NEJ, eds. The new Production of Users: Changing Innovation Collectives and Involvement Strategies, London: Routledge; 2016.

[4] Hinman LM, ed. Contemporary Moral Issues. Diversity and Consensus. London: Routledge; 2010.

[5] Boenink M, Van Lente H, Moors E, eds. Emerging Technologies for Diagnosing Alzheimer’s Disease. Innovating with Care. Palgrave Macmillan; 2016.

[6] Cuijpers Y, van Lente H (2015). Early diagnostics and Alzheimer’s disease: beyond ‘cure’ and ‘care. ’ Technol Forecast Soc Change.

[7] Brown N (2015). Metrics of hope: Disciplining affect in oncology. Health.

